# Supplementary material for: Prioritizing sequence variants in conserved non-coding elements in the chicken genome using chCADD
Source: PLoS Genet. 2020 Sep 23;16(9):e1009027. doi: 10.1371/journal.pgen.1009027 (PMC7535126; doi:10.1371/journal.pgen.1009027)
Supplement: S1 Fig — The scale is adjusted to make the differences between the models visible. Penalization of 1 was selected due to the lowest log-loss and largest ROC-AUC. (PDF) [file pgen.1009027.s001.pdf]

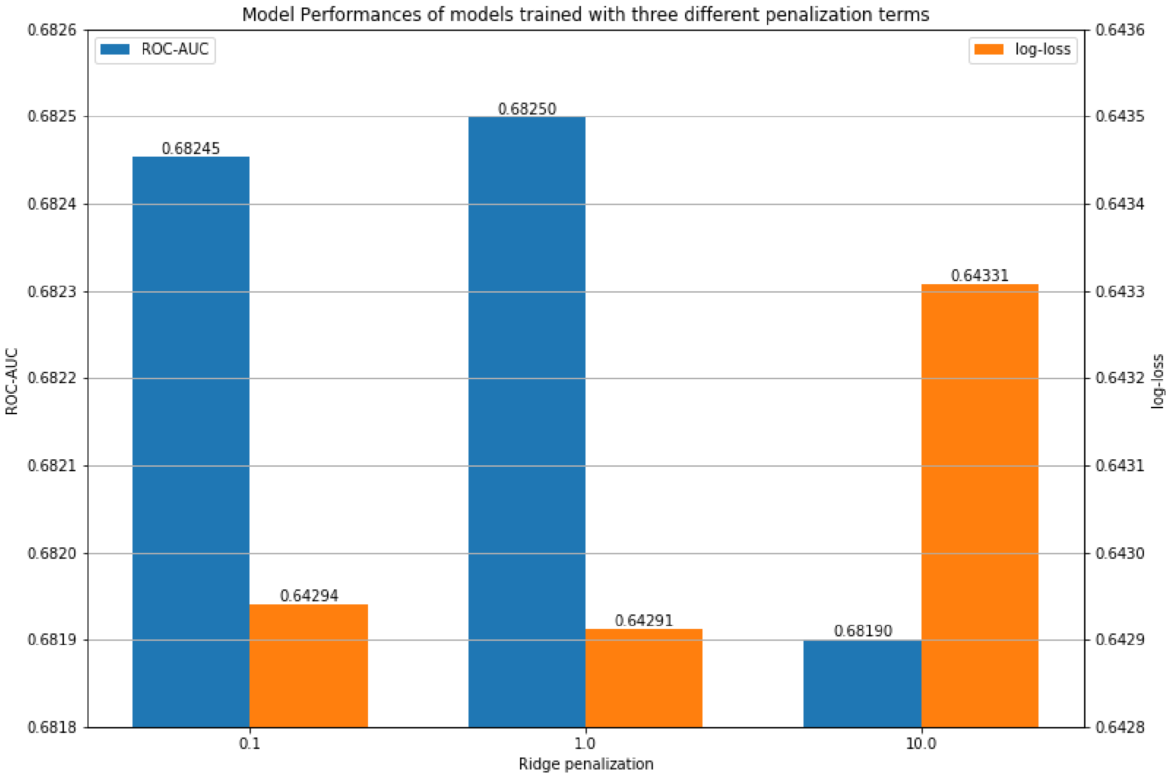

S1 Fig. Model performances measured in Receiver Operator Area under the Curve (ROC-AUC) and log-loss for three different ridge penalization terms (0.1, 1.0, 10.0). The scale is adjusted to make the differences between the models visible. Penalization of 1 was selected due to the lowest log-loss and largest ROC-AUC.
