# Supplementary material for: Prioritizing sequence variants in conserved non-coding elements in the chicken genome using chCADD
Source: PLoS Genet. 2020 Sep 23;16(9):e1009027. doi: 10.1371/journal.pgen.1009027 (PMC7535126; doi:10.1371/journal.pgen.1009027)
Supplement: S2 Fig — The barplot displays the fraction of the genome per chromosome covered by conserved elements. (PDF) [file pgen.1009027.s002.pdf]

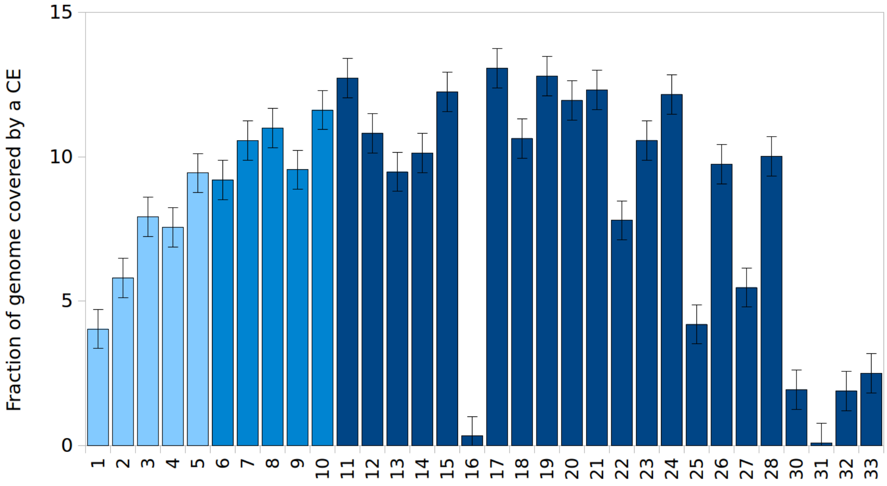


**S2 Fig. Distribution of conserved elements (CEs) along the chicken genome.** The barplot displays the fraction of the genome per chromosome covered by conserved elements.
