# Supplementary material for: Prioritizing sequence variants in conserved non-coding elements in the chicken genome using chCADD
Source: PLoS Genet. 2020 Sep 23;16(9):e1009027. doi: 10.1371/journal.pgen.1009027 (PMC7535126; doi:10.1371/journal.pgen.1009027)
Supplement: S3 Fig — The y-axis shows the frequency, while the x-axis the size in base pairs (bp) of the predicted conserved elements. (PDF) [file pgen.1009027.s003.pdf]

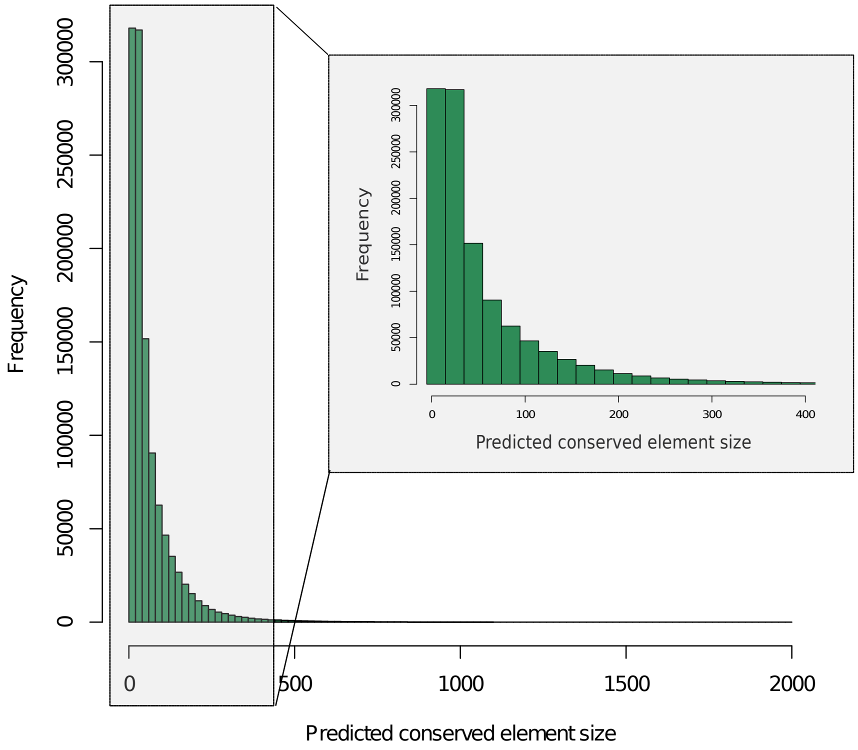

S3 Fig. Frequency size distribution of predicted conserved elements. The y-axis shows the frequency, while the x-axis the size in base pairs (bp) of the predicted conserved elements.
