# Supplementary material for: Prioritizing sequence variants in conserved non-coding elements in the chicken genome using chCADD
Source: PLoS Genet. 2020 Sep 23;16(9):e1009027. doi: 10.1371/journal.pgen.1009027 (PMC7535126; doi:10.1371/journal.pgen.1009027)
Supplement: S4 Fig — The exonic-associated conserved elements include CDS, 5’UTR, 3’UTR, and promoter regions. (PDF) [file pgen.1009027.s004.pdf]

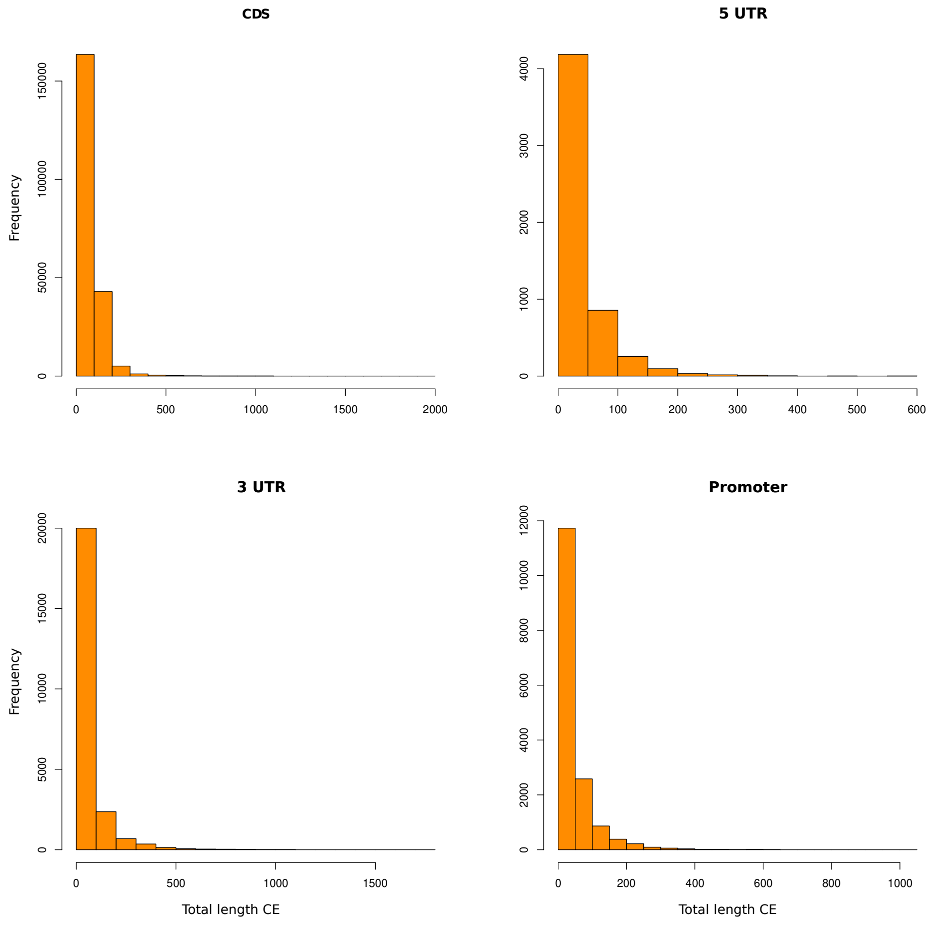

S4 Fig. Frequency size distribution of predicted conserved elements overlapping exonic-associated gene annotations. The exonic-associated conserved elements include CDS, 5'UTR, 3'UTR, and promoter regions.
