# Supplementary material for: Prioritizing sequence variants in conserved non-coding elements in the chicken genome using chCADD
Source: PLoS Genet. 2020 Sep 23;16(9):e1009027. doi: 10.1371/journal.pgen.1009027 (PMC7535126; doi:10.1371/journal.pgen.1009027)
Supplement: S5 Fig — The non-protein-coding gene annotations include introns, lncRNA, and intergenic regions. (PDF) [file pgen.1009027.s005.pdf]

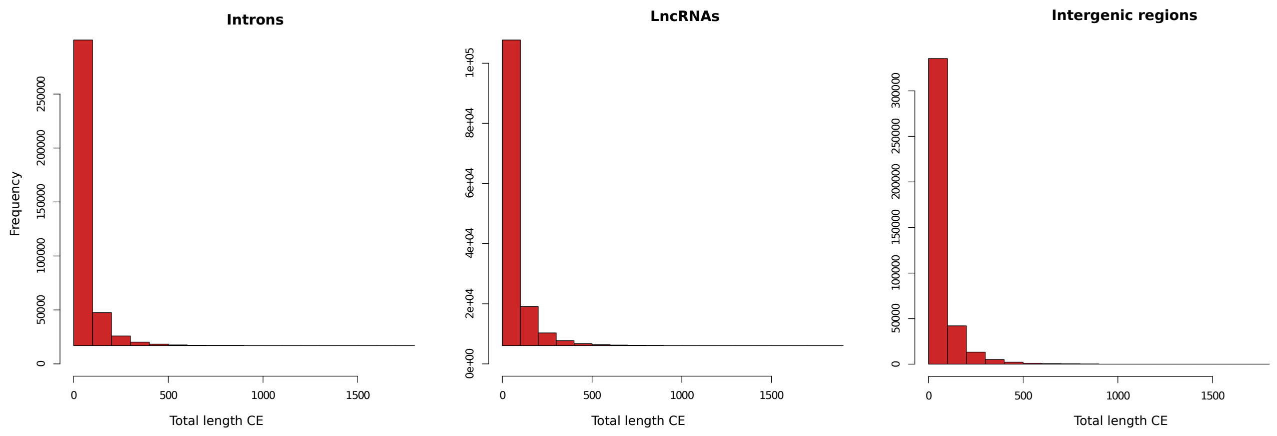

S5 Fig. Frequency size distribution of predicted conserved elements overlapping non-protein-coding gene annotations. The non-protein-coding gene annotations include introns, lncRNA, and intergenic regions.
