# Supplementary material for: Prioritizing sequence variants in conserved non-coding elements in the chicken genome using chCADD
Source: PLoS Genet. 2020 Sep 23;16(9):e1009027. doi: 10.1371/journal.pgen.1009027 (PMC7535126; doi:10.1371/journal.pgen.1009027)
Supplement: S1 Table — Missing values are imputed via the specified values. Annotations of the type (factor) are OneHotEncoded and combinations between annotations form the final feature set. (PDF) [file pgen.1009027.s006.pdf]

S1 Table. List of annotations which form the set of descriptive features for which model weights are learned. Missing values are imputed via the specified values. Annotations of the type (factor) are OneHotEncoded and combinations between annotations form the final feature set.

Annotation label	Data type	Imputed value	Annotation description
Ref	factor	-	Reference allele
Alt	factor	-	Observed allele
isTv	bool	0.5	Is transversion?
Consequence	factor	-	VEP Consequence summaries
GC	num	0.4	Percent GC in a window of +/- 75bp
CpG	num	0.02	Percent CpG in a window of +/- 75bp
motifECount	int	0.0	Total number of overlapping motifs
motifEHIPos	bool	False	Is the position considered highly informative for an overlapping motif by VEP
motifEScoreChng	num	0.0	VEP score change for the overlapping motif site
Domain	factor	UD	Domain annotation inferred from VEP annotation (ncoils, tmhmm, sigp, lcompl, ndomain = "other named domain")
Dst2Splice	int	0.0	Distance to splice site in 20bp; positive: exonic, negative: intronic
Dst2SplType	factor	UD	Closest splice site is ACCEPTOR or DONOR
oAA	factor	UD	Amino acid of observed variant
nAA	factor	UD	Reference amino acid
Grantham	int	0.0	Grantham score: oAA,nAA
SIFTcat	factor	UD	SIFT category of change
SIFTval	num	0.0	SIFT score
cDNApos	int	0.0	Base position from transcription start
relcDNApos	num	0.0	Relative position in transcript
CDSpos	int	0.0	Base position from coding start
relCDSpos	num	0.0	Relative position in coding sequence
protPos	int	0.0	Amino acid position from coding start
relProtPos	num	0.0	Relative position in protein codon
dnaRoll	num	0.23	Predicted local DNA structure effect on dnaRoll
dnaProT	num	0.68	Predicted local DNA structure effect on dnaProT
dnaMGW	num	0.03	Predicted local DNA structure effect on dnaMGW
dnaHelT	num	-0.12	Predicted local DNA structure effect on dnaHelT
GerpS	num	-0.17	Rejected Substitution' score defined by GERP++
GerpN	num	0.64	Neutral evolution score defined by GERP++
GerpRS	num	0.0	Gerp element score
GerpRSpval	num	1.0	Gerp element p-Value
4PhCons_noChick	num	0.17	4-taxa-sauropsids PhastCons score (excl. chicken)
37PhCons_ noChick	num	0.13	37-taxa-Amniota PhastCons score (excl. chicken)
77PhCons_ noChick	num	0.2	77-taxa-Vertebrate PhastCons score (excl. chicken)
4PhyloP_ noChick	num	0.07	4-taxa-sauropsids PhyloP score (excl. chicken)
37PhyloP_ noChick	num	0.04	37-taxa-Amniota PhyloP score (excl. chicken)
77PhyloP_ noChick	num	0.25	77-taxa-Vertebrate PhyloP score (excl. chicken)
minDistTSS	int	10000000	Distance to closest Transcribed Sequence Start (TSS)
minDistTSE	int	10000000	Distance to closest Transcribed Sequence End (TSE)
interaction-score	num	0	Interaction score from Hi-C interaction maps
Exp-score	int	0	RNA expression scores
Exp-pval	num	1	p-Value of RNA expression scores
Exp-logFC	num	0	Log-Fold change of RNA expression
OChrom-Peaknb	Int	0	Read number for open Chromatin; ATAC-seq
OChrom-pval	num	1	p-Value for open chromatin; ATAC-seq
OChrom-logFC	num	0	Log-Fold change for ATAC-seq
