# Supplementary material for: Prioritizing sequence variants in conserved non-coding elements in the chicken genome using chCADD
Source: PLoS Genet. 2020 Sep 23;16(9):e1009027. doi: 10.1371/journal.pgen.1009027 (PMC7535126; doi:10.1371/journal.pgen.1009027)
Supplement: S2 Table — (PDF) [file pgen.1009027.s007.pdf]

S2 Table. GO term enrichment analysis of exonic-associated CE and intronic CEs.

Term ID	Term description	Target size	3' UTR				Intron			
			Term size	Query size	Overlap	p-Value	Term size	Query size	Overlap	p-Value
GO:0048856	Anatomical structure development	12,514	3,293	4,736	1,475	$1.24x10^{-17}$	3,293	6,971	2,128	$1.09x10^{-29}$
GO:0010646	Regulation of cell communication	12,514	2,038	4,736	917	$3.67x10^{-09}$	2,038	6,971	1,329	1.33^{-17}
GO:0010604	Positive regulation of macromolecule metabolic process	12,514	2,118	4,736	952	$1.49x10^{-09}$	2,118	6,971	1,331	$2.21x10^{-09}$
GO:0023051	Regulating of signaling	12,514	2,056	4,736	926	$2x10^{-09}$	2,056	6,971	1,339	$1.88x10^{-17}$
GO:0048583	Regulation of response to stimulus	12,514	2,332	4,736	1,032	$1.44x10^{-08}$	2,332	6,971	1,477	$9.79x10^{-13}$
GO:0048468	Cell development	12,514	1,364	4,736	625	$1.27x10^{-06}$	1,364	6,971	927	$1.12x10^{-18}$
GO:0031325	Positive regulation of cellular metabolic process	12,514	2,091	4,736	936	$9.01x10^{-09}$	2,091	6,971	1,304	$1.09x10^{-07}$

Term ID	Term description	Target size	CDS				5' UTR			
			Term size	Query size	Overlap	p-Value	Term size	Query size	Overlap	p-Value
GO:0048856	Anatomical structure development	12,514	3,293	9,703	2,713	$2.06x10^{-11}$	3,293	1,896	654	$5.13x10^{-14}$
GO:0010646	Regulation of cell communication	12,514	2,038	9,703	1,686	$2.64x10^{-06}$	2,038	1,896	381	$9.33x10^{-03}$
GO:0010604	Positive regulation of macromolecule metabolic process	12,514	2,118	9,703	1,749	$3.53x10^{-06}$	2,118	1,896	403	$5.06x10^{-04}$
GO:0023051	Regulating of signaling	12,514	2,056	9,703	1,699	$4.46x10^{-06}$	2,056	1,896	384	$9.24x10^{-03}$
GO:0048583	Regulation of response to stimulus	12,514	2,332	9,703	1918	$5.55x10^{-06}$	2,332	1,896	424	$4.39x10^{-02}$
GO:0048468	Cell development	12,514	1,364	9,703	1,142	$1.78x10^{-05}$	1,364	1,896	282	$3.38x10^{-05}$
GO:0031325	Positive regulation of cellular metabolic process	12,514	2,091	9,703	1,723	$1.91x10^{-05}$	2,091	1,896	388	$1.60x10^{-02}$
