# Supplementary material for: Prioritizing sequence variants in conserved non-coding elements in the chicken genome using chCADD
Source: PLoS Genet. 2020 Sep 23;16(9):e1009027. doi: 10.1371/journal.pgen.1009027 (PMC7535126; doi:10.1371/journal.pgen.1009027)
Supplement: S3 Table — If multiple annotations exist for the same variant, the consequence is selected according to the displayed hierarchy, starting at 1 and ending at 14. (PDF) [file pgen.1009027.s008.pdf]

S3 Table. VEP consequences summarized in 14 categories. If multiple annotations exist for the same variant, the consequence is selected according to the displayed hierarchy, starting at 1 and ending at 14.

**Hierarchy**	**Abbreviation**	**VEP Consequence**
1	SG	Stop Gained
2	CS	Canonical Splice
3	NS	Non-Synonymous
4	SN	Synonymous
5	SL	STOP Lost
6	S	Splice Site
7	U5	5'-UTR
8	U3	3'-UTR
9	IG	Intergenic
10	NC	Noncoding-change
11	I	Intronic
12	UP	Upstream
13	DN	Downstream
14	O	Unknown / Other
