# Supplementary material for: Prioritizing sequence variants in conserved non-coding elements in the chicken genome using chCADD
Source: PLoS Genet. 2020 Sep 23;16(9):e1009027. doi: 10.1371/journal.pgen.1009027 (PMC7535126; doi:10.1371/journal.pgen.1009027)
Supplement: S4 Table — (PDF) [file pgen.1009027.s009.pdf]

S4 Table. Top 10 model features with the largest assigned weight and their explanations.

Label	Model weight assigned to feature	Feature explanation
GerpS	0.152568	GERP rejected substitution score
4PhCons_noChick	0.28726	4-sauropsids PhastCons scores (excluding chicken)
I_GerpS	0.109099	GERP rejected substitution score for intronic sites
I_4PhCons_noChick	0.0899441	4-sauropsids PhastCons scores (excluding chicken) for intronic sites
dnaProT	0.083813	DNA secondary structure prediction for ProT
77PhCons_noChick	0.0790709	4-amniota PhastCons scores (excluding chicken
dnaRoll	0.0733429	DNA secondary structure prediction for Roll
IG_4PhCons_noChick	0.067539	4-sauropsids PhastCons scores (excluding chicken) for intergenic sites
I_dnaProT	0.0671401	DNA secondary structure prediction for ProT for intronic sites
IG_GerpS	0.0635293	GERP rejected substitution score for intergenic sites
