# Supplementary material for: Prioritizing sequence variants in conserved non-coding elements in the chicken genome using chCADD
Source: PLoS Genet. 2020 Sep 23;16(9):e1009027. doi: 10.1371/journal.pgen.1009027 (PMC7535126; doi:10.1371/journal.pgen.1009027)
Supplement: S5 Table — Differences are measured in absolute Cohen’s D between the different subregions in which each CNEs was subdivided in the change point analysis. (PDF) [file pgen.1009027.s010.pdf]

S5 Table. Differences between genomic annotations utilized for the chCADD model. Differences are measured in absolute Cohen's D between the different subregions in which each CNEs was subdivided in the change point analysis.

**Intronic**	**UP-1st**	**1st-2nd**	**2nd-3rd**	**3rd-Down**
4PhastCons	594	307	361	609
37PhastCons	446	328	369	448
77PhastCons	1.25	96	195	1.32
4PhyloP	0.43	0.09	126	428
37PhyloP	351	187	214	0.35
77PhyloP	776	186	237	778
GerpS	272	182	196	257
GerpN	212	112	0.11	214
dnaMGW	103	9	7	104
dnaProT	0.08	13	12	0.08
dnaHelT	82	2	2	83
GC	121	45	47	0.12
CpG	34	34	34	34
OChrom-Peaknb	58	1	91	15
OChrom-logFC	62	87	138	17
OChrom-pval	6	13	70	55
**LncRNA**	**UP-1st**	**1st-2nd**	**2nd-3rd**	**3rd-Down**
4PhastCons	608	289	338	623
37PhastCons	469	0.31	342	482
77PhastCons	1.29	86	184	1.37
4PhyloP	428	83	117	0.43
37PhyloP	343	161	0.18	348
77PhyloP	788	0.17	0.22	792
GerpS	267	0.17	181	259
GerpN	212	86	98	201
dnaMGW	97	6	8	95
dnaProT	96	9	9	93
dnaHelT	89	3	0.0	86
GC	114	37	41	109
CpG	24	33	29	28
OChrom-Peaknb	59	-0.02	64	23
OChrom-logFC	102	93	137	55
OChrom-pval	12	96	103	5
**Intergenic**	**UP-1st**	**1st-2nd**	**2nd-3rd**	**3rd-Down**
4PhastCons	0.61	281	341	619
37PhastCons	474	319	359	481
77PhastCons	1.29	84	179	1.37
4PhyloP	431	84	119	432
37PhyloP	351	162	185	351
77PhyloP	0.79	167	215	795
GerpS	0.29	169	183	274
GerpN	209	91	88	215
dnaMGW	96	8	8	96
dnaProT	97	14	12	96
dnaHelT	86	3	2	84
GC	136	62	62	136
CpG	39	37	36	41
OChrom-Peaknb	17	4	0.02	5
OChrom-logFC	89	5	12	77
OChrom-pval	0.00	5	52	23
