# Supplementary material for: Prioritizing sequence variants in conserved non-coding elements in the chicken genome using chCADD
Source: PLoS Genet. 2020 Sep 23;16(9):e1009027. doi: 10.1371/journal.pgen.1009027 (PMC7535126; doi:10.1371/journal.pgen.1009027)
Supplement: Attachment — Submitted filename: Rebuttal.pdf [file pgen.1009027.s013.pdf]

We would like to thank the Associate Editor and Editor-in-Chief of PLOS Genetics for allowing us to revise our manuscript. We would also like to thank the three (anonymous) reviewers for their comments and for recognizing the high importance and scientific impact of the developed ch(icken)CADD score in the field of chicken genomics. In this rebuttal letter, we have addressed all concerns and recommendations expressed by the reviewers point by point. All changes made to the text are in red to facilitate the reviewing process. We have also changed the e-mail address of one of our corresponding authors. Compared to the initial submission, we believe that our current revised manuscript has considerably improved and is now suitable to be considered for publication in PLOS Genetics.

Reviewer's Responses to Questions
Comments to the Authors:
Please note here if the review is uploaded as an attachment.

Reviewer #1:
This is a valuable contribution to chicken genomics as the authors have established a database with CADD scores in an attempt to predict the functional significance of sequence variants in the chicken genome. The analysis appears solid and the data are in general well presented. I have the following comments:
We would like to thank the reviewer for recognising the importance and contribution of the ch(icken)CADD score developed in our study to the scientific, and particularly chicken, community. We have addressed all comments point by point below.

1. The title can be improved, I recommend to delete "harbour functionally important variation" because this is the obvious and most common explanation for evolutionary conservation. This study does not provide any functional assessment of non-coding variants so the title becomes misleading. It is highly appropriate to include chCADD in the title
We agree with the reviewer and changed the original title of the manuscript to "Prioritizing sequence variants in conserved non-coding elements in the chicken genome using chCADD"

2. Abstract middle and in several other places: I am missing something after (chCADD) for instance "scores" or "database".
We thank the reviewer for pointing this out. We added the word 'model' (see comment of reviewer #3) since in the abstract we refer to CADD as a variant effect prediction tool.

3. As far as I understand the Author Summary should be popular science-style which is not the case here. For instance, "a change point analysis" is not a common term for laymen.
We removed jargons and also abbreviations from the Author Summary. We have also added two more sentences (in red in the text) to make the Author Summary more accessible to the lay public.

4. Page 3. Please add a brief explanation how many and which type of breeds were included in the analysis.
We have added further details on the number and type of chicken breeds used in the study, see Line 67-68 of the Materials and methods ("The dataset comprised a total of 169 individuals sampled 67 from 88 traditional chicken breeds of divergent demographic and selection history").

Line 272. The title is misleading. It would be more appropriate to say something like "CNEs are less common in gene dense regions"
We agree and have changed it according to the reviewer's recommendation (see Line 277).

Line 285-286, the same number of decimal places should be used here and it would be easier for the readers if you write that the SNP density is two-fold higher in non-CNEs (=0.02) than in CNEs (=0.01).

General: It is not clear what you mean by SNP density and you should use average nucleotide diversity instead. This take into account SNP density and allele frequency spectra and should be more powerful for this analysis.
We changed our sentence as suggested by the reviewer (Line 293-294). However, we did not replace the SNP density with the nucleotide diversity for the following reason. In this study, we defined SNP density as the number of single nucleotide polymorphisms identified in the 169 birds divided by the number of bases found in the sequence (Line 227-228). In line with previous studies in humans (Drake et al. 2006), plants (i.e. Haudry et al. 2013), and chicken (i.e. Seki et al. 2017), conserved regions are conserved because of selective constraints. Although mutations can occur in these regions, they are usually actively selected against. We therefore expect a lower than expected SNP density in CNEs than in non-CNEs and a derived allele frequency (DAF) significantly shifted towards the ancestral allele (as we here showed). We here wanted to separately investigate whether the SNP density and the DAF followed the expected pattern instead of looking at their combined effect in the form of nucleotide diversity.

Line 301. It is more appropriate to write "the lower proportion" as the difference is small.
We changed the sentence (see Line 309).

Line 310. "chCADD scores potentially causal variants higher". Higher compared with what?? My suggesting is to show the distribution of CADD scores for coding and non-coding regions separately in a main figure. Then the authors can judge how extreme the scores presented in Table 3 are. Furthermore, the subtitle is not optimal since the verb scores could be misinterpreted as a substantive.
We agree with the reviewer. We changed the title of the subsection to ***chCADD distinguishes between potentially causal and non-causal variants (****Line 327-328). Further, we added two more boxplots to the supplementary figure 6 which display the distribution of chCADD scores of SNPs located in coding and non-coding regions. We included supplementary figure 6 in the main text as figure 3.*

Table 3. It is sufficient to use one decimal place for the CADD scores.
We agree with the reviewer. We use a one decimal place to present the results consistently throughout the manuscript (please refer to Table 3 on page 11/21).

Figure 1. I think that Figures 1-3 can be merged into one figures with three panels.
We decided to keep Figure 1 as a standalone figure, because we had to add an extra analysis and thus figure to figure 2 (see comment reviewer #3). We therefore decided to combine Figure 2 with Figure 3.

Figure 3. Is the relatively high frequency of derived SNPs that are fixed an artefact or is it just a consequence of drift and derived alleles reaching a "point of no return"?
Our investigated chicken population consists of domesticated species in which drift effects play a major role in these breeding circumstances. It is therefore not suprising to observe a high proportion of derived fixed variants. Similar findings were also reported in (Derks et al. 2018 and Bortoluzzi et al. 2020).*

**Derks, M.F., Megens, H.J., Bosse, M., Visscher, J., Peeters, K., Bink, M.C., .. & Groenen, M.A. (2018). A survey of functional genomic variation in domesticated chickens. Genetics Selection Evolution, 50(1), 1-14; Bortoluzzi, C., Bosse, M., Derks, M.F., Crooijmans, R.P., Groenen, M.A., & Megens, H.J. (2020). The type of bottleneck matters: insights into the deleterious variation landscape of small managed populations. Evolutionary applications, 13(2), 330-341.*

Figure 5. This figure should be based on nucleotide diversity rather than SNP density. A consistent number of decimal places should be used in the figure. The much higher SNP density 5bp downstream than 5bp upstream must be an artefact that the authors need to correct or explain properly.

For an explanation on the use of SNP density rather than nucleotide diversity, please refer to our answer to the comment above. We followed the advice of the reviewer and changed the picture and show all numbers with 1 decimal only. The reason why there are relatively more SNPs in the 5 bp-downstream region than in the upstream region is also not completely clear to us.

First, we thought that it might be an artefact by PhastCons and its 2-state phylo-HMM model. The 5-bp-downstream region represents the first 5 bp after the switch from the conserved to the nonconserved state for which it may need a minimum of phylogenetic signal which would make the model flip to the nonconserved state and such signal would be given by a slightly bit more variable site in the multiple sequence alignment (MSA). To test this hypothesis, we downloaded data for the tutorial example 3 provided on the PhastCons website (http://compgen.cshl.edu/phast/phastCons-tutorial.php) and then created a reversed fasta file using the BioPython SeqIO module. Following, we created phastcons scores for the forward fasta and reversed fasta file and plotted these results (Fig. 1). To validate that these are the same distributions, only horizontally reversed, we flipped the reversed distribution in via the vector graphics program InkScape and overlayed them (reversed distribution red opaque, original blue opaque). If there would be differences in PhastCons scores for a particular site, depending if the previous site is already conserved, than these two distributions would differ slightly but they perfectly overlap each other.

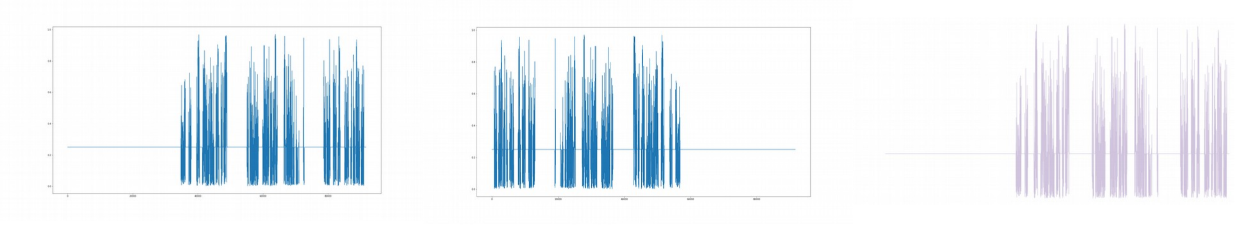

(a) (b) (c)

Figure 1: PhastCons scores for (a) forward fasta sequences, (b) reversed fasta sequences, and (c) both distributions overlayed to each other to see if there are any differences. Original blue opaque, reversed, red opaque.

We then decided to test if the upstream and downstream regions of our CNE blocks are differing in any particular feature which may explain the differences in observed SNPs. For that we annotated these regions for their difference in GC, CpG and open chromatin identified through ATAC-Seq. The differences in these features (Table 1) between the upstream and downstream regions are marginally different from 0 and considering the standard deviation not the reason why we see a consistent higher number of SNPs in the downstream region than upstream.

Table 1: Results for mean differences between up and downstream regions and their standard deviations.

Intron	***Mean diff up & down***	***Std diff up & down***
GC	*~2.5*10^-5*	*~0.04*
CpG	*~6*10^-5*	*~0.02*
OChrom	*0*	*0*

Intergenic	***Mean diff up & down***	***Std diff up & down***
GC	*~0.01*	*~0.06*
CpG	*~0.01*	*~0.03*
OChrom	*0*	*0*

lncRNA	***Mean diff up & down***	***Std diff up & down***
GC	*~1.3*10^-5*	*~0.05*

CpG	*~-7*10^-5*	*~-7*10^-5*
OChrom	*0*	*0*

We further checked if the annotation of SNPs to these different regions might be wrong but we did not identify any bug in our code as well. Concluding, we do not have a satisfying explanation for this difference. We clarified this in the main text (Line 384-386).

Reviewer #2:
This study was to develop chCADD to identify conserved non-protein-coding regions (CNE) with functionally important variants in chicken genome. The predicted CNEs with potential functional constraint based on chCADD could be useful in narrowing down candidate SNPs for causative mutations in chickens. However, current chCADD model is still suffering from not integrating other important epigenomic data available in chickens (although it is limited compared to human and mouse) as epignomic information could provide more weighted information on the CNE. This is particularly true from the recent 2019 CADD paper (CADD: predicting the deleteriousness of variants throughout the human genome. Philipp Rentzsch et al. Nucleic Acids Research, Volume 47, Issue D1, 08 January 2019, Pages D886–D894).
In addition, several recent studies indicated a considerable CNEs, especially enhancers, might be missed based on sequence conservation across evolutionarily distant species, as they are not necessarily conserved in sequence, but potentially functionally conserved on the regulatory role of the regions across multiple species.
We thank the reviewer for the comments and suggestions. We agree with his/her comment on the lack of extensive functional, and particularly epigenomic data in the chCADD model. We do use the publicly available epigenomic data released by the FAANG consortium (Table S1), but we noted that we neglected to mention this explicitly in the main text. We have remedied this (Line 180-181). More generally, however, note that meaningful models which outperform individual conservation scores genome-wide can be generated with a restricted set of features, to which epigenomic data adds little in terms of performance, as recently highlighted in mouse by us.*

Groß, C., de Ridder, D. & Reinders, M. Predicting variant deleteriousness in non-human species: applying the CADD approach in mouse. BMC Bioinformatics* ***19,* *373 (2018)*

From Table 3, it seems if the phenotype is not deleterious, chCADD score for regulatory variants are very low. does this mean the prediction would not be accurate as SNPs in the regulatory regions in general are not detrimental, but could be functionally important for economically important traits.
We agree with the reviewer's comments that if a SNP is not detrimental to survival, it may not have a high score. This is due to the fact that the phylogenetic signal to score SNPs with respect to their predicted deleteriousness is obtained over evolutionary time scales while especially economical traits have been particularly under selection relatively recently. In other words, what has been deleterious in the past may not be deleterious nowadays anymore and vice versa. On the other hand, mutations that disturb biological processes have a higher chance to cause an effect on the phenotype and those are picked up by the score, if this change of phenotype is of economical interest or just detrimental for the individual has to be decided by the breeder. In other words, the chCADD scores are helpful to order sets of SNPs with respect to their potential interest, but for any final decision with regard to their importance on a trait, further information should be acquired. We discuss this issue further in the Discussion (Line 527-532).

The mspt could be improved by comparing annotated regulatory regions from recent published FAANG data in chickens with CNEs identified in this study.

We already used epigenetic data, ATAC-seq and Hi-C, as well as RNA-seq expression data in the creation of the chCADD model (see above) as obtained from the FAANG consortium. We believe that using regions derived from the same data for comparison may lead to biased results and circular reasoning.

Minor comments: current Ensembl version is 99, not sure why release 95 was used (Line 230). From Line 82, not sure why using galGal4 as more updated version galGal6 is available.
We used Ensembl release 95 for two main reasons. First, the 4 sauropsids EPO alignment used for the reconstruction of the ancestral sequence was downloaded immediately after the Ensembl release 95. Therefore, for consistency reasons, we used data and tools from the same Ensembl release throughout the analyses. Second, since the release of the new chicken genome assembly with the Ensembl release 95, data involving chicken, including the 4 EPO alignment, have not been updated. Therefore, we do not expect major changes between release 95 and release 99.
As discussed in Lines 83-95, we had to anchor the 23 sauropsids multiple sequence alignment to galGal4 because the chicken genome assembly used in the alignment was galGal4. However, we converted all genomic coordinates in the multiple alignment file (MAF) to the most recent genome assembly (GRCg6a) to avoid any misinterpretation in our results. We performed a similar step when looking at the variants present in the OMIA database (Line 198-199). Therefore, all results presented in the manuscript are reported in the context of the most recent chicken genome assembly.

Reviewer #3:
In their manuscript "Evolutionarily conserved non-protein-coding regions in the chicken genome harbor functionally important variation" (PGENETICS-D-20-00791) Christian Groß and others describe an analysis of non-protein coding regions in the chicken genome. They use conserved elements identified from multi-species whole genome alignments and they explore how conservation scores or combined annotation scores can be used to break down larger conserved elements into potential functional units. As part of these efforts, the authors release a "Combined Annotation Dependent Depletion" (CADD) model for chicken with this publication. For those unfamiliar, CADD was originally developed for the human genome, where a rich set of available annotation can be combined in a model trained on human derived fixed changes (that were exposed to many generations of purifying selection) vs. simulated "de novo" variants (that have not seen purifying selection). Several of the authors were involved in two previous publications that have extended the development of CADD scores to mouse and pig. While mouse again profits from being a model species, the pig model – similar to the proposed chicken model – needs to rely heavily on gene model derived annotations and species conservation. Many epigenetic features and genomic segmentations are simply unavailable. This is a "the best that we can do" situation and from earlier results as well as the ones presented here, the combined score performs better than just using a single (conservation) metric. Preparing and maintaining scores for three species is also a substantial effort and provides a relevant resource for the respective communities. All three score sets currently provided are limited to SNVs (already in training). Therefore, authors provide scores for all SNVs rather than tools to calculated mCADD, pCADD or chCADD scores.

I only have a number of small comments that I would like the authors to address:

Line 75: What are these filter criteria? Is that what is described in "Genome-wide distribution and density of conserved non-protein-coding regions" it needs a cross reference.
We thank the reviewer for pointing this out. The filtering criteria we used in the generation of the VCF file are different from the ones used to filter our variants after annotation with VEP. We added a few more lines in the methodology about our settings (Line 75-81). This information will be useful for reproducibility purposes.

Line 134: Not sure, I understand the intronic EST filter. Please explain what you are addressing here.

We realized that the way our filtering step is presented is misleading. We rephrased this and added a reference to the study we based it on (Lines 140-141).

Line 135: How can the protein-coding variants fall outside of coding sequence?
We thank the reviewer for pointing out this error, we removed this sentence.

Line 163-164: Is that a different alignment than one used above? Why?
The alignment is the same but the second time we spelled out the individual species in that alignment. We see that this is confusing, therefore we changed the order and explain the content of the 4 EPO sauropsidis alignment first and then just refer to the 4 EPO alignment afterwards.

Line 177: "imputed" - reference Table S1 for values.
We added the reference to Table S1 to the position in the text.

Line 187: Feature scaling. Your scaling as described is alright, just wondering whether you also mean-centered and forgot to mention. Mean centering would only help if one wanted to interpret the model offset, so it is not relevant but commonly done. Also, have you considered releasing the determined SDs and scripts for scoring variants? You might also consider releasing scripts for annotating, deriving training data and scoring variants; in case you are not planning to commit on future score releases (e.g. due to updated reference sequences). Releasing the obtained scores (as already done) is critical; releasing more is of course optional.
We only scaled the data columns using SDs, without mean centering because we did not investigate the individual parameters any further. Besides of the scores, we have made a Python script available to annotate SNPs with the chCADD scores, the fine-mapped CNE blocks, a script to re-create the SNP density bar plots from Figure 5. We released files containing the imputation and scaling values for each feature, scripts to create and apply these on a data set, the trained model, a scaled train and test set, the model coefficients in a text file, and files containing the original SNPs and the SNPs at each processing and annotation step. Further, scripts which do the SNP annotation and ReadMe.txt files describing the files and scripts in each directory.

Line 274-275: I am trying to wrap my head around that. All originate from a distribution of conserved elements. Wondering whether a random distribution from which you subtract the coding, would always result in a negative correlation with coding, in which case this would probably need a different test.
For this analysis, we divided the genome in sections of 100kb and over the set of these sections, we found a negative correlation between the density of (Figure 2a in the main text). We performed a similar analysis, but this time we scaled the CNE count within each window to the remaining sequence length after substracting the coding sequence. We then correlated once again the density of CNEs with that of exons (Figure 2b in the main text) and also observed a negative correlation, although this correlation was slightly smaller than that in Figure 2a.

Figures: Huge issues in PDF conversion. Linked PNGs are much better, but have issues with transparency (tested several programs and did not find one that shows it correctly).
For the current resubmission we uploaded our figures to the PACE digital diagnostic tool. We hope that all figures are now of higher quality in order to be accurately assessed by the reviewer.

Textual edits:

Abstract: "Depletion (chCADD) [annotation|model],"

Changed to model.

Author summary: "The chCADD [model] assign[s] a score"
Changed.

Line 55: "Depletion (chCADD) model, in the tradition"
Changed.

Line 100: "falling [in] or overlapping"
Changed.
